# COVID-19 course in granulomatosis with polyangiitis: single center experience with review of the literature

**DOI:** 10.55730/1300-0144.5389

**Published:** 2022-03-05

**Authors:** Berkan ARMAĞAN, Mehmet Akif EKSİN, Serdar Can GÜVEN, Bahar ÖZDEMİR, Pınar AKYÜZ DAĞLI, Orhan KÜÇÜKŞAHİN, Ahmet OMMA, Abdulsamet ERDEN

**Affiliations:** 1 Rheumatology Clinic, Ankara City Hospital, Ankara, Turkey; 2 Division of Rheumatology, Department of Internal Medicine, Ankara Yıldırım Beyazıt University, Ankara, Turkey

**Keywords:** Coronavirus disease 2019, severe acute respiratory syndrome coronavirus 2, granulomatosis with polyangiitis, Wegener’s granulomatosis, rituximab

## Abstract

**Background/aim:**

Coronavirus disease 2019 (COVID-19) shares some clinical features with new-onset granulomatosis with polyangiitis (GPA) or GPA flare that may lead to a challenge in differential diagnosis. To date, little is known whether GPA can be induced by COVID-19. Herein, we aimed to seek the frequency and mortality rates of COVID-19 in our GPA cohort, and along with the literature cases, to evaluate clinical features and treatments of GPA patients with COVID-19. We also tried to identify clinical features of COVID-19 induced GPA.

**Materials and methods:**

As of July 2021, we conducted a systematic literature review using different spelling combinations of “COVID-19 and GPA” in the PUBMED database. In total, 18 cases were found in the literature, 6 of them had COVID-19 induced GPA. The remaining 12 of literature cases and 6 cases in our GPA cohort (n = 81) had a COVID-19 infection while followed-up with GPA. We grouped these 18 patients as GPA+COVID-19.

**Results:**

The frequency of COVID-19 was 7.4% in GPA cohort and mortality rate was 33% in GPA patients with COVID-19. The most common symptoms of GPA+COVID-19 patients were fever, cough, arthralgia/myalgia, and malaise. The most frequent treatments for GPA before the COVID-19 infection were steroids (72%) and rituximab (56%). Three patients who received rituximab also had COVID-19 reinfection. In the literature cases, mortality was observed in 4 (22%) of 18 patients with GPA+COVID-19. The most common symptoms of COVID-19 induced GPA were dyspnea, fever and cough.

**Conclusion:**

In our GPA cohort, we observed a higher mortality rate compared to global WHO data. In patients followed up with GPA, rituximab treatment may be precarious for both COVID-19 disease and reinfection. Our study also provided some clues about the diagnostic challenge of GPA induced by COVID-19.

## 1. Introduction

Coronavirus disease 2019 (COVID-19), which is caused by severe acute respiratory syndrome coronavirus 2 (SARS-CoV-2), could be mostly mild, but some patients could develop severe disease with pneumonia, acute respiratory failure and sepsis which may lead to mortality. In COVID-19, approximately 80% of cases are mild, 15% severe and 5% critical [1, 2]. Advanced age, male sex, and comorbidities such as obesity, diabetes mellitus, cardiac and pulmonary diseases are the poor prognostic factors for COVID-19 [3]. SARS-CoV-2 induces viral pyroptosis (severe inflammatory programmed cell death) in infected respiratory epithelial cells, like other cytopathic viruses. That cell/tissue destruction may trigger a subsequent inflammatory response In fact, in most patients, the immune system suffices to eliminate or limit this process. However, in some patients inflammation, because of a dysfunctional immune response^1^, could not be limited and a cytokine storm is triggered [4].

Granulomatosis with polyangiitis (GPA) is a systemic, necrotizing vasculitis mediated by antineutrophil cytoplasmic antibodies (ANCA) against proteinase 3 (PR3). The respiratory manifestations of GPA may be mild, similar to many viral respiratory tract infections with malaise, arthralgia and respiratory tract symptoms, or it may be characterized by more severe lower respiratory tract symptoms and even acute respiratory failure because of alveolar hemorrhage and may cause mortality [6–9].

In the literature, the variable clinical course of COVID-19 is also reported in patients with autoimmune rheumatic diseases (ARD). Ultimately, there are not enough studies with a high level of evidence to enable us to evaluate the course of SARS-CoV-2 infection, especially in the rare ARDs like vasculitides [9–13]. According to various case series, active rheumatological disease, organ involvement, and some immunosuppressive therapies such as rituximab and glucocorticoid (especially prednisolone >10 mg/day or equivalent dose) could lead to a worse course of COVID-19 disease. On the other hand, the effects of other antirheumatic drugs on COVID-19 course are not clear. Even, the inflammatory processes associated with SARS-CoV-2 infection could be restricted to some immunosuppressive agents by the effect of suppressing proinflammatory cytokine pathways [14–16]. Antibody-associated immunity is one issue recently examined in COVID-19. Although the effect of B-cell-depleting rituximab on viral clearance is not yet clear. These agents might alter COVID-19 elimination rates, disease course or prognosis [17, 18]. Since various immunosuppressives, including B-cell depleting agents, are used in GPA, it is important to evaluate the course of COVID-19 in this patient group. Additionally, the differential diagnosis of all ANCA-related vasculitis (AAV) from the COVID-19 is challenged due to similar clinical, laboratory, and radiological features. To date, GPA has only been presented as COVID-19 induced or coinfection in few case reports [10, 19, 20].

Therefore, we aimed to evaluate the clinical course, mortality of COVID-19 in our GPA patients with cases compiled in the literature.

## 2. Material and method

As of July 2021, we conducted a systematic literature review using a combination of the terms “COVID-19” or “Covid-19” or “covid-19” or “COVID 19” or “Covid 19” or “covid 19” with “Granulomatosis with polyangiitis” or “Wegener’s granulomatosis” or “ANCA associated vasculitis” or “GPA” in the PUBMED database. In the included publications, language was limited to “English”. While preparing this article, the articles on patients with GPA who had COVID-19 infection or who were diagnosed COVID-19 infection induced GPA, together with the different definitions above, were taken into consideration.

The literature review was carried out by MAE, SCG and all authors worked in collaboration in selecting and obtaining the data. The study protocol was approved by the local ethics committee (E2-21-173). In the selection process, first the title and abstract of the articles were evaluated. In this evaluation, 54 articles were found that could be related to our subject (Figure). In total, 19 of these articles were about patients with GPA who had COVID-19 or GPA induced by COVID-19. All GPA patients were meeting the American College of Rheumatology diagnostic criteria [21]. After all, 18 case reports including 18 patients’ full texts of which could be accessed, were evaluated within the scope of our study. A total of 12 GPA cases who had COVID-19 were presented as case report in the literature to date [6, 22–32]. The remaining 6 cases were diagnosed with GPA following COVID-19 and presented as COVID-19 induced in the literature [19, 20, 33–36]. Thus, we also grouped these patients as COVID-19 induced GPA patients. Besides 12 cases from the literature [6, 22–32], COVID-19 was detected in 6 patients in our GPA cohort. These18 patients were grouped as GPA+COVID-19. In total, 24 patients (18 GPA+COVID-19 and 6 COVID-19 induced GPA) were included in this study.

Demographics, clinical characteristics, comorbidities, organ involvements and treatments of GPA and ANCA serology of our patients were evaluated with the cases in the literature. Enzyme-linked immunosorbent assay (ELISA) and immunofluorescent antibody assay (IFA) methods were used to detect perinuclear (p-ANCA) or cytoplasmic (c-ANCA) ANCA staining and for proteinase 3 (PR3-ANCA) or myeloperoxidase (MPO-ANCA) antibody positivity, respectively. Besides these data, clinical features, organ involvements of COVID-19, hospitalization, length of hospitalization, requirement for systemic glucocorticoids or oxygen support and mortality due to COVID-19 were also recorded. All systemic glucocorticoid doses, including prednisolone and deflazacort used by the patients, were recorded as methylprednisolone equivalent. Birmingham Vasculitis Activity Score (BVAS) before COVID-19 infection and Vasculitis Damage Index (VDI) and Revised Five Factor Score (FFS) scores after COVID-19 infection were also evaluated [37–39]. Pre- and post-COVID-19 BVAS scores were calculated using the prescribed scoring algorithm and grouped as remission, persistent and new/worse [39, 40]. COVID-19 diagnosed in our GPA cohort by positive nasal swab reverse transcriptase–polymerase chain reaction (RT-PCR) or compatible thorax computed tomography (CT) findings accompanied by clinical symptoms of COVID-19. We searched all our GPA patients’ medical records in the hospital database and contacted them via telephone.

Statistical analysis was performed using the Statistical Package for the Social Sciences version 22 (SPSS v.22). The variables were investigated using visual (histograms, probability plots) and analytical methods (Shapiro–Wilk’s test) to determine whether or not they are normally distributed. Descriptive analyses were presented using tables of frequencies for the ordinal variables and using medians and interquartile range (IQR) or minimum-maximum (min-max) for the nonnormally distributed mean and standard deviation, as indicated. Categorical variables are presented as percentages. The Mann–Whitney U test or Student’s t-test were used for comparison of continuous variables, according to normality of distribution. For the evaluation of categorical variables, the Pearson’s chi-squared test and Fisher’s exact test were used, as indicated. The Wilcoxon test was used to compare the change in BVAS score between pre- and post-COVID-19 disease. For all comparisons, p values <0.05 were considered statistically significant.

## 3. Results

### 3.1 COVID-19 and GPA outcomes

COVID-19 was seen in 6 (7.4%) of the 81 GPA patients under follow-up in our cohort. A total of 18 patients (12 GPA patients from the literature and 6 from our cohort) were grouped as GPA+COVID-19. The median (min-max) age of 18 GPA+COVID-19 patients was 55.6 (21–88) years. Demographic, clinical and laboratory characteristics of GPA+COVID-19 patients were shown in Table 1. The most common comorbidity was hypertension (n = 7, 39%). The most common symptom of COVID-19 was fever (67%) and at least half of the patients had cough, fatigue and arthralgia/myalgia. Eleven patients (61%) had both RT-PCR positivity and an appearance compatible with COVID-19 on thorax CT, and 5 (29%) patients had only RT-PCR positivity (thorax CT of 3 patients was not compatible with COVID-19 and 2 patients did not have any data about pulmonary imaging). In 2 (12%) patients with a history of contact with a SARS-CoV-2 positive individual, COVID-19 diagnosed based on typical pulmonary radiological and clinical findings. At the time of COVID-19 diagnosis, 14 patients (78%) were using glucocorticoids due to GPA, of which 8 were using low-dose, 3 medium-dose and 2 high-dose. Glucocorticoid dose in one of the literature cases had not been reported. Patients were compared according to prednisone use prior to COVID-19 infection. There was no difference in terms of oxygen requirement, hospitalization length, ICU admission, and mortality between patients who received any dose of prednisone and those who did not. In total, 10 patients (59%) received rituximab and 9 patients received cyclophosphamide therapy due to GPA. Two of them were under active cyclophosphamide induction regimen for GPA at the time of COVID-19 diagnosis. There were 8 patients who received rituximab therapy in the last 12 months before COVID-19 diagnosis, while the time between the last rituximab infusion and the development of COVID-19 was unknown in 2 patients. Apart from these, azathioprine was administered to 4 patients (24%), methotrexate to 2 (12%), and mycophenolate mofetil to 1 (6%). During COVID-19, it was determined that 7 (39%) of 18 patients needed oxygen support.

Comparison of our and literature cases in terms of BVAS scores before and after COVID-19 was shown in Table 2 and VDI and revised FFS scores before COVID-19 infection was shown in Table 3. The median (min-max) BVAS score was 1 (0–11) before COVID-19, and 1 (0–6) after COVID-19 (p = 0.273). Detailed clinical features of all GPA patients with COVID-19 disease were presented in Table 4.

### 3.2 GPA and COVID-19 reinfection

Among GPA+COVID-19 patients, COVID-19 reinfection was reported in 3 patients (2 females and 1 male). These were cases in the literature and were receiving rituximab. Besides rituximab treatment, one of these patients was taking prednisone (30 mg/day) plus cyclophosphamide, and the other was taking only prednisone 5 mg/day. Oxygen support was needed in 2 patients, but no mortality was observed in these 3 patients. All patients had stable disease BVAS scores before and after COVID-19 reinfection. The time interval between two courses of COVID-19 was as follows for the 3 cases: after 43 days with 2 negative PCR control, 47 days with 1 negative PCR control and after six months with 3 negative PCR control and seroconversion.

### 3.3 COVID-19 related mortality in GPA patients

In our GPA cohort, COVID-19 mortality was 33% (n = 2/6). Along with literature cases, mortality was observed in 4 (22%) of 18 GPA+COVID-19 patients (Table 1). Comparison of deceased and surviving GPA+COVID-19 patients was similar in terms of age, sex, pulmonary and renal involvement due to GPA, median steroid dose, rituximab usage, and pulmonary involvement in thoracic CT due to COVID-19 (Table 5). Of the patients who died, 2 were receiving rituximab (1 patient from our cohort) and 2 glucocorticoid alone. Although all deceased patients had a higher median (min-max) revised FFS and VDI score before COVID-19 disease, there was no statistically significant difference [2 (2–2) vs. 1 (0–2), p = 0.073 and 2 (0–6) vs. 0 (0–6), p = 0.310, respectively]. The median BVAS values of all patients before COVID-19 disease were 0 (0–3), while it was 1 (0–11) in survivors (p = 0.611).

### 3.4. COVID-19 induced GPA

There were 6 cases diagnosed with GPA after COVID-19 infection, all of which were reported in the literature. Demographic and clinical characteristics of these patients were shown in Table 6. The time interval between COVID-19 and diagnosis of GPA ranged from 10 days to 1 month for all 6 cases. Of these patients, c-ANCA positivity was found in 5 and PR-3 ANCA in 1, and also pulmonary involvement in thoracic CT due to GPA in 3, renal involvement due to GPA in 5 (4 necrotizing glomerulonephritis). In these COVID-19 induced GPA patients, the most common COVID-19 symptom was fever with 66%. In comparison to GPA+COVID-19 patients, COVID-19 induced GPA patients had a higher cough, dyspnea and a lower fatigue and arthralgia/myalgia incidence. Of these patients, 83% had ground-glass opacities on thorax CT, and 66% had RT-PCR positivity. Oxygen support was needed in 2 patients and mortality was observed in 1 patient.

## 4. Discussion

The frequency and mortality of COVID-19 were 7.4% and 33% in our GPA cohort, respectively. Among the literature cases, the most common symptoms of GPA+COVID-19 cases were fever, cough, arthralgia/myalgia, and malaise. The most frequent treatments for GPA before the COVID-19 infection were prednisone (72%) and rituximab (56%). Three patients receiving rituximab also had COVID-19 reinfection. Mortality due to COVID-19 was observed in 4 (22%) of 18 GPA+COVID-19 patients. The most common symptoms of COVID-19 induced GPA were dyspnea in addition to those common in GPA+COVID-19, such as fever and cough.

In our current literature review, there was no study on the incidence of COVID-19 in GPA patients. AAVs were only 2% in the European League Against Rheumatism (EULAR) COVID-19 database with 4373 cases registered^2^. In total, 7.4% of GPA patients in our cohort had COVID-19 disease and the mortality rate was 33%. According to WHO COVID-19 dashboard, global mortality rates of COVID-19 was 2.13% at the same time as our study^3^. We observed a higher mortality rate in GPA patients with COVID-19 in our study. According to the COVID-19 Global Rheumatology Alliance (GRA) Physician-Reported Registry, COVID-19-related mortality among patients with rheumatic disease is associated with advanced age, male sex, certain comorbidities and disease-specific factors like disease activity and certain medications [13]. The course of the COVID-19, especially in patients receiving rituximab, could be severe [13, 41, 42]. In a multicenter study including diverse rheumatological diseases, mortality was observed in 7 of 17 AAV patients with COVID-19, and it was reported that rituximab was used in 4 of these 7 patients [43]. Rituximab is a biological agent that binds to CD20 on the surface of B lymphocyte cells and inhibits antibody development [44]. These potential deteriorating effects of rituximab on COVID-19 disease course could be likely due to these alterations in the humoral immune system. Although rituximab use was similar between deceased and surviving GPA+COVID-19 patients in our study, it was shown that rituximab therapy was used in 60% of 18 patients and in all patients who developed COVID-19 reinfection. Therefore, these data suggest that rituximab therapy may be a negative factor for COVID-19 disease prognosis once again. Apart from rituximab, it has also been reported in various case reports that the use of glucocorticoids (≥10 mg/day prednisone) increases the hospitalization rate and mortality risk due to COVID-19 disease [13, 27, 45, 46]. In our study, comparison of the GPA+COVID-19 patients with and without prednisone use at any dose, oxygen requirement, length of hospitalization, ICU admission rate, and mortality was similar. According to the COVID-19 GRA physician-reported registry data, other factors associated with COVID-19-related mortality in patients with rheumatic disease were disease activity and organ damage [13]. Although there was no statistical difference in terms of VDI and revised FFS scores between the deceased GPA+COVID-19 patients and survivors, these scores were higher in GPA+COVID-19 deceased patients in our study.

COVID-19 induced GPA has been reported in 6 patients with respect to literature data. The frequency of fever and cough was similar between GPA+COVID-19 and COVID-19 induced GPA patients. But, in comparison to GPA+COVID-19 patients, COVID-19 induced GPA patients had higher frequency of dyspnea and lower frequency of fatigue and arthralgia/myalgia. According to these findings, in patients with constitutional symptoms predominantly other than fever, COVID-19 could be considered more prioritized in the differential diagnosis of GPA. In a patient presenting with similar symptoms, if pulmonary complaints such as cough and dyspnea are more predominant from fatigue and arthralgia/myalgia, which are non-specific findings, GPA should always be considered, except for COVID-19 disease. In the light of all this limited and speculative data, COVID-19 disease should be kept in mind in the differential diagnosis of GPA and vice versa.

This has been the first study about the incidence of COVID-19 in GPA patients in single-center. However, there were some limitations. Most importantly, it has been a retrospective study and the number of patients was little. In addition, the literature case reports were from different centers and the lack of clinical and laboratory data of them (especially the presence of hypogammaglobulinemia for patients receiving rituximab) were other limitations of this study. RT-PCR is repeated 2–3 times every other day for our patients with RT-PCR negativity but with a high suspicion of COVID-19, but there was no clear data on what was done in these situations in the literature cases. Last, the status of GPA at the COVID-19 disease, and also the information on whether mortality due to COVID-19 disease or post-COVID vasculitis activation is not clear.

In conclusion, the frequency of COVID-19 in our GPA cohort was 7.4% and the mortality rate due to COVID-19 was higher. In patients followed up with GPA, rituximab treatment may be precarious for both COVID-19 disease and reinfection. Finally, a patient presenting with GPA findings should also be examined for COVID-19 disease. To obtain more accurate results about COVID-19 disease in GPA patients, more comprehensive studies with more patients are needed.

## Figures and Tables

**Figure:**
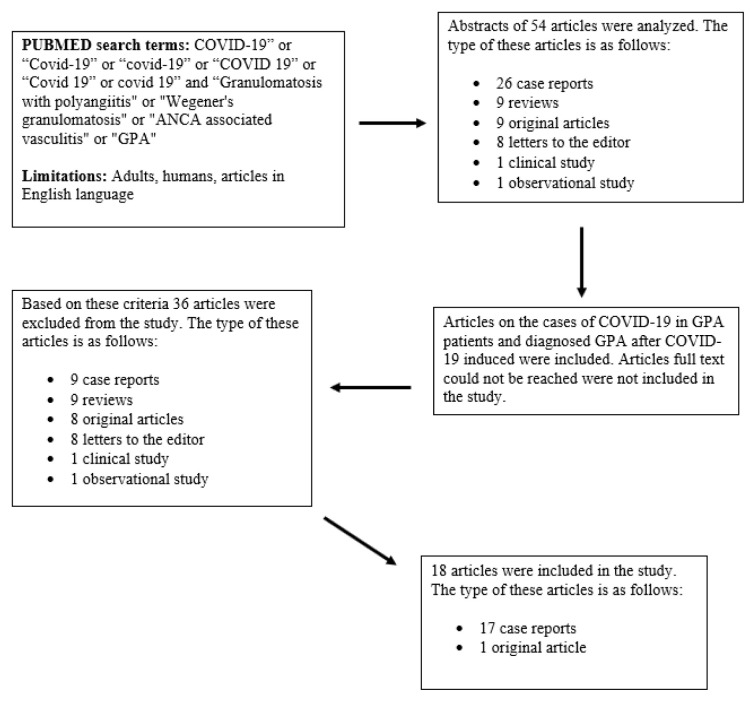
Flow chart of article selection process

**Table 1:** Comparison of demographic, clinical and laboratory characteristics of GPA+COVID-19 cases between literature and our cases.

	GPA+COVID-19 cases		
	Literature cases (n = 12)	Our cases (n = 6)	Total (n = 18)
**Median age, years (min-max)**	53.5 (21–80)	62.5 (43–88)	57.0 (21–88)
**Male, n (%)**	7 (58)	4 (67)	11 (61)
**Female, n (%)**	5 (42)	2 (33)	7 (39)
**Comorbidity, n (%)**			
*Hypertension*	3 (25)	4 (67)	7 (39)
*Chronic kidney disease*	2 (17)	2 (33)	4 (22)
*Hyperlipidemia*	2 (17)	2 (33)	4 (22)
*Obesity*	1 (8)	1 (17)	2 (11)
*Coronary artery disease*	0	1 (11)	1 (6)
*Diabetes mellitus*	1 (8)	0	1 (6)
*Osteoporosis*	1 (8)	0	1 (6)
**COVID-19 symptoms, n (%)**			
*Fever*	8 (67)	4 (67)	12 (67)
*Cough*	9 (75)	1 (17)	10 (56)
*Fatigue*	4 (33)	5 (83)	9 (50)
*Arthralgia/Myalgia*	4 (33)	5 (83)	9 (50)
*Dyspnea*	4 (33)	3 (50)	7 (39)
*Anorexia*	1 (8)	4 (67)	5 (28)
*Sore throat*	2 (17)	1 (17)	3 (17)
*Nausea*	0	3 (50)	3 (17)
*Vomiting*	0	2 (33)	2 (11)
*Ageusia*	2 (17)	0	2 (11)
*Rhinitis*	1 (8)	1 (7)	2 (11)
*Headache*	1 (8)	1 (17)	2 (11)
*Diarrhea*	1 (8)	1 (17)	2 (11)
*Hemoptysis*	2 (17)	0	2 (11)
*Fever with chills*	1 (8)	1 (17)	2 (11)
*Sputum*	1 (8)	0	1 (6)
*Anosmia*	1 (8)	0	1 (6)
**RT-PCR positivity, n (%)**	12 (100)	4 (67)	16 (89)
**Lymphopenia, n (%)**	4 (33)	6 (100)	10 (56)
**Ground glass opacitiesin thorax CT, n (%)**	9 (75)	4 (67)	13 (72)
**C-Reactive protein (>5 mg/L), n (%)**	7 (58)	4 (67)	11 (61)
**Need for O** **_2_** ** support, n (%)**	4 (33)	3 (50)	7 (39)
**Immunosuppressive usage for GPA, n (%)**			
*Prednisone*	10 (83)	4 (67)	14 (78)
*Rituximab*	9 (75)	1 (17)	10 (56)
*Cyclophosphamide*	1 (8)	1 (17)	2 (11)
*Mycophenolate mofetil*	1 (8)	0	1 (6)
**Mortality, n (%)**	2 (17)	2 (33)	4 (22)

RT-PCR: Reverse transcriptase–polymerase chain reaction, CT: Computed tomography, GPA: Granulomatosis with polyangiitis.

**Table 2:** Comparison of the pre-COVID-19 and post-COVID-19 Birmingham Vasculitis Activity Scores of our and literature GPA+COVID-19 cases.

	Our cases*		Literature cases**		Total	
BVAS	Pre-COVID-19	Post-COVID-19	Pre-COVID-19	Post-COVID-19	Pre-COVID-19	Post-COVID-19
**Remission**	**40**	**25**	**45**	**33**	**44**	**31**
**Persistent symptoms**	**40**	**75**	**37**	**45**	**37**	**54**
**New/worse symptoms**	**20**	**0**	**18**	**22**	**19**	**15**

BVAS: Birmingham Vasculitis Activity Scores.

* The BVAS score of pre-COVID-19 in 1 and post-COVID-19 in 2 (due to mortality) were not available in our cases.

** The BVAS score of pre-COVID-19 in 1 and post-COVID-19 in 3 (2 of them due to mortality) were not available in the literature cases.

**Table 3:** Comparison of pre-COVID-19 vasculitis damage index and revised five factor scores between our and literature GPA+COVID-19 cases.

	Literature cases* (n = 11)	Our cases (n = 6)	Total (n = 17)

Revised FFS, median (min-max)	2 (0–2)	2 (0–2)	2 (0–2)

Revised FFS scores, n (%)			
0	3 (27)	1 (17)	4 (22)
1	2 (18)	3 (17)	3 (17)
2	6 (55)	4 (66)	10 (56)

VDI, median (min-max)	0 (0–2)	1 (0–6)	0 (0–6)

* Revised FFS and VDI scores were not available for one literature case. All comparisons between our cohort and literature were statistically insignificant.

FFS: Five factor score, VDI: Vasculitis damage index.

**Table 4:** Detailed clinical features of granulomatosis polyangiitis patients with COVID-19 disease.

		Granulomatosis with polyangiitis related				COVID-19 disease related			
Patient no.	Age (years), sex	Major organ involvement	Duration (years)	Treatments	Last dose of RTX	Pneumonia	Treatments	Hospitalization	Mortality
1	66, F	Pulmonary	4	MTX MPZ 4 mg		No	No	No	No
2	71, M	Pulmonary Renal	2	MPZ 4 mg		Yes	Azithromycin Favipiravir MPZ PEX LMWH	Yes	Yes
3	43, M	Pulmonary Renal	3	AZA 50 mg MPZ 4 mg		No	Favipiravir	No	No
4	59, M	Neuropathy	4	RTX AZA MPZ 4 mg	4 months	Yes	Favipiravir MPZ Pulse IVIg	Yes	Yes
5	45, F	Renal	1	AZA		Yes	LMWH	Yes	No
6	88, M	Pulmonary	1	CYC		Yes	HCQ	Yes	No
*7*	21, M	Pulmonary	3	MMF 3 gr MPZ 24 mg		Yes	Remdesivir DXM 8 mg CPT LMWH	Yes	No
*8*	40, M	None	2 months	MTX MPZ		Yes	None	Yes	No
*9*	37, F	Pulmonary	15	MPZ Pulse PEX		No	TCZ	Yes	Yes
*10* *	77, M	Pulmonary Pachymeningitis	18	RTX MPZ 32 mg	1 month	Yes	Azithromycin	Yes	No
							Remdesivir MPZ 12 mg CPT		
*11* *	30, F	None	NA	RTX	2.5 months	NA	None	NA	No
							Bamlanivimab		
*12*	35, E	Pulmonary	7	RTX AZA MPZ 6 mg	NA	Yes	Azithromycin HCQ	Yes	No
*13*	63, M	Renal	7	RTX MPZ 4 mg	14 days	Yes	None	Yes	No
*14*	77, F	None	3	RTX MPZ 4 mg	1 month	Yes	NA	Yes	No
*15* *	61, F	Pulmonary Renal	1	RTX CYC MPZ 24 mg	5 months	NA	None	Yes	No
							DXM LMWH		
*16*	55, M	Pulmonary Renal Pachymeningitis	27	RTX MPZ 4 mg	5 months	Yes	Azithromycin HCQ Lopinavir/ritonavir	Yes	No
*17*	80, M	Renal	7	RTX	NA	Yes	MPZ PEX	Yes	Yes
*18*	52, F	Pulmonary	33	RTX MPZ 12 mg	7 days	Yes	Lopinavir/ritonavir HCQ	Yes	No

* Cases with COVID-19 reinfection. In COVID-19 specific treatment squares, the top line shows the drugs used for the initial infection, and the bottom line shows the drugs used for reinfection.

RTX: Rituximab, MTX: Methotrexate, MPZ: Methylprednisolone, PEX: Plasmapheresis, LMWH: Low molecular weight heparin, AZA: Azathioprine, IVIg: Intravenous immune globulin G, CYC: Cyclophosphamide, HCQ: Hydroxychloroquine sulfate, MMF: Mycophenolate mofetil, DXM: Dexamethasone, CPT: Convalescent plasma therapy, NA: Nonavailable, TCZ: Tocilizumab.

**Table 5:** Comparison of demographic, clinical characteristics and treatments between deceased and surviving GPA+COVID-19 cases.

	Deceased (n = 4)	Surviving (n = 14)	Total (n = 18)	p value
**Median age, years (SD)**	61.8 (18.6)	53.8 (19.4)	55.6 (19.0)	0.477
**Male, n (%)**	3 (75)	8 (57)	11 (61)	0.486
**Pulmonary involvement, n (%)**	1/3 (3)	9/13 (69)	9/16 (56)	0.304
**Renal involvement, n (%)**	2/3 (67)	5/12 (42)	7/15 (47)	0.538
**Ground glass opacitiesin thorax CT, n (%)**	3/4 (75)	10/11 (91)	13/15 (87)	0.476
**Prednisonedosage, mg/day, median (min-max)**	4 (−)	6.2 (4–40)	5 (15)	0.364
**Rituximab, n (%)**	2/3 (67)	8/13 (62)	10 (63)	0.696

GPA: Granulomatosis with polyangiitis, CT: Computed tomography.

**Table 6:** Demographic, clinical and laboratory characteristics of COVID-19 induced GPA cases.

	Total, (n = 6)
**Median age, years (min-max)**	42 (25–60)
**Male, n (%)**	3 (50)
**Symptom, n (%)**	
*Fever*	4 (66)
*Cough*	4 (66)
*Dyspnea*	3 (50)
*Hemoptysis*	2 (33)
*Fatigue*	2 (33)
*Chest pain*	1 (17)
*Arthralgia/Myalgia*	1 (17)
*Anorexia*	1 (17)
*Rhinitis*	1 (17)
**RT-PCR positivity, n (%)**	4 (66)
**Lymphopenia, n (%)**	1 (17)
**Ground glass opacities in thorax CT, n (%)**	5 (83)
**C-reactive protein (>5 mg/L), n (%)**	3 (50)
**Need for O** **_2_** ** support, n (%)**	2 (33)
**Mortality, n (%)**	1 (17)

GPA: Granulomatosis with polyangiitis, RT-PCR: Reverse transcriptase–polymerase chain reaction, CT: Computed tomography.

## Footnotes

1 SSRN (2020). Cell Pyroptosis, a Potential Pathogenic Mechanism of 2019-nCoV Infection [Online]. Website https://ssrn.com/abstract=3527420 [accessed 01 July 2021].

2 EULAR COVID-19 Database (2021). European paediatric and adult database [Online]. Website https://www.eular.org/myUploadData/files/eular_covid_19_registry_report_20210201.pdf [accessed 01 July 2021].

3 World Health Organization (2021). WHO Coronavirus (COVID-19) Dashboard [Online]. Website https://covid19.who.int/info [accessed 01 July 2021].
